# On Nervous Palpitation of the Heart and Its Treatment

**Published:** 1886-09

**Authors:** 


### *ON NERVOUS PALPITATION OF THE HEART AND ITS TREATMENT.*

The editors of the *Therapeutic Gazette* do not hesitate to exceptionally devote the columns intended for brief and condensed abstracts of foreign therapeutic matters to full translations, if they meet with an article combining scientific interest with practical worth to so eminent a degree as in the case of Dr. Skorczewski's paper, appearing under the above heading in the April issue of the Vienna *Zeitschrift für Therapie.* The paper, with omission of some unimportant statistical matters, reads as follows:

"Nervous palpitation of the heart is an affection which develops without any visible and, moreover, without any anatomical causes, and is based upon the very disagreeable sensation of an exceptionally prominent and augmented cardiac action. The elder writers comprised, under this category, all morbid processes characterized by an increased heart's action, while at present we exclude all affections not referring to a purely nervous cause but to anatomical alterations, and certain special lesions known as angina pectoris and Basedow's disease. After this exclusion we have left the genuine nervous palpitation known also as cardiopalmus cardiagmus.

"The augmented cardiac action is caused by a functional interference with its nerves, which, in its turn, may result either from an irritation of the cardiac accelerator (the sympathetic) nerve, or from a paralysis of the cardiac inhibitory nerve (pneumogastric), or any point of its course from the centres in the spinal cord to the centres in the heart. But it is very difficult, and sometimes impossible, to determine the cause of the palpitation and the anatomical site of the cause.

"In spite of the frequency of nervous palpitation, few authors have gathered pertinent statistical materials. In my practice I found the palpitation in 6.4 per cent. of all patients, but much more frequently in women than in men (proportion 8 to 1).

"The palpitation appears with a varying frequency in various ages. In females I have often noticed it already between the fifth and the tenth year (1.6 per cent.), and between the tenth and fifteenth year (2.1 per cent.). Most frequently, however, the affection appears between the fifteenth and twentieth year (20 per cent.), then between the twenty-first and thirtieth year (18.3 per cent.). Between the thirtieth and fortieth year the percentage falls to 9.7, between forty-one to fifty to 3.7, and beyond the fiftieth year we find but very few cases (1 per cent).

"In men the affection does not appear before the age of twenty, and remains stationary regarding frequency up to the forty-fifth year; later it becomes very rare (0.5 per cent.)

"Chlorosis and all the blood dyscrasias play a very important role in the development of nervous palpitation in a similar manner, as has been supposed in the case of Basedow's disease (Friederich, Grafe, Romberg, Taylor). In the cases coming under my observation 76 per cent. showed a simultaneous dyscrasia of the blood, 26 per cent. chlorosis; 35 per cent. anæmia; 36 per cent. malarial intoxication. Texta believes that in malaria the palpitation refers directly to the engorgement of the spleen, though my own observations have not confirmed this view.

"Various authors, such as Traube, Henoch, Murchison, and Frerichs, have called attention to the frequent connection of nervous palpitation with digestive troubles, a view which my own observations have confirmed.

"Botkin found the cause in one case of nervous palpitation in the so-called wandering kidney. In 101 cases of wandering kidney treated by me I observed nervous palpitation in 10.8 per cent.

"In affections of the genital tract I noted nervous palpitation in 12.1 per cent., and especially in structural alterations, such as new formations, flexions, versions, and the products of inflammation. In dysmenorrhœa and menorrhagia nervous palpitation is a common complication.

"In nervous troubles the palpitation appears especially frequently, (86 per cent.). The following specification throws an interesting light upon this connection: General neuroses—neurasthenia, spinal irritation, hysteria, hypochondria, 77.8 per cent.; megrim, 27.0 per cent.; intercostal neuralgia, 10 2 per cent.; neuralgia of the pneumogastic nerve and myalgia, 4.3 per cent.

"The clinical appearances of nervous palpitation are very varying. The patients describe the sensation in different manners. Some are unable to describe it at all, others complain of a trembling of the heart or of powerful movements, after which the muscle appears to cease working. Sensations of pain and pressure are almost universally admitted. Being asked to localize their sensations, the patients again give wholly different answers. Some point to the region of the left side of the heart, others to the middle of the chest or to the entire left side, others, again, to different extremities. Some describe their sensations as proceeding from the heart and wandering over the whole left side of the chest, and, occassionally, the palpitation is said to be felt most on the neck or along the spinal column. The palpitation paroxysms differ also in the same person in duration and frequency. Preisendörfer observed paroxysms, lasting several hours, and Gerhardt, some lasting several days.

"In the cases coming under my observation I noted three different groups: 1. Those with an augmented apex-beat; 2. Those with an increased frequency of the beat; 3. Those without any alteration of the heart's action.

"The clinical course of the palpitation is usually parallel with that of the affections which have caused it, the palpitation remaining, in exceptional cases, only after the disappearance of its causative agencies. In such cases our affection calls, of course, for a special treatment. A long duration of the palpitation may give rise to serious troubles, and predisposes to anatomical alterations.

"The therapeutics of nervous palpitation, when resulting from other primary troubles, must first be directed to the removal of these. It is especially important to do away with any existing abuse of liquids, such as coffee, tea and alcoholic drinks, and to observe strict dietetic and hygienic precautions. Indulgence in smoking is particularly objectionable. The affections of blood-dyscrasia (chlorosis, anæmia, malaria), of the nervous, digestive, and genital apparatus, call, of course, for an especial treatment. For the removal or lessening of the intensity of the paroxysms various methods of treatment have been recommended.

" Preisendörfer advocates pressure upon the pneumogastric nerve, while Mühlberger recommends massage of the epigastric region, together with derivative drugs.

" Schröter employed, successfully, cold or ice applications to the heart, and Rosenbach ether locally for the same purpose. Digitalis, which is recommended by Schröter, is wholly disapproved of by Leyden in this affection, also in Basedow's disease.

" Leyden claims that narcotics, especially morphine, given internally, or better, hypodermically, give gratifying results in the paroxysms of angina pectoris, based upon sclerosis of the coronary arteries, and Rosenbach and Schröter obtained good results with these remedies, also, in nervous palpitation. Chloral, chloroform and ether also have the reputation of favorably influencing the paroxysms. The first remedy is to be given internally, the two others, both as inhalations, and internally in large doses (½ to 1 teaspoonful).

" Rosenbach recommends, also, bromide of potassium in large doses (30 to 45 grains) and iodoform (3 to 7 grains). Nitrite of amyl, first introduced seventeen years ago by Lauder Brunton, enjoys still an excellent reputation. Hay recommends this remedy, and also nitrite of ethyl, nitrite of methyl, and particularly nitrite of sodium.

" Hammond, Hay, and Green speak also well of very minute doses of nitro-glycerin (1 to 3 to 10 drops of a one per cent. solution).

" Omitting all other drugs, which, though recommended from various physicians, have never attained any popular hold in the treatment of palpitation, I proceed to allude to the results which the above remedies have given in my hands.

" Pressure upon the pneumogastric nerve, massage, and cold applications appeared in no way to favorably influence the affection. Morphine, though a great stand-by in all painful troubles, is not well borne by patients of the nervous and hysterical type, and therefore its exhibition in palpitation is little recommendable. Nitrite of amyl and nitro-glycerin give but doubtful results when first exhibited, and scarcely any later. The following measures constitute my routine treatment. During the paroxysm of the palpitation I order cold applications to the region of the heart, provided they are—what is not always the case—well borne by the patient. Besides, I prescribe antispasmodic remedies, among which I prefer a solution of bromide of sodium, 15 to 30 grains, to be given either at once or in two doses. I also give the patient aqua laurocerasi, with equal parts of tincture of valerian (or of tinct. cann. indic. or tinct. castorei sibirici), to be taken in doses of 5 to 10 drops every few minutes. In frequently returning paroxysms I order a powder, consisting of bromide of sodium (7 grains) and quinine (1½ to 3 grains), to be taken three times daily before each meal, and Fowler's solution (5 to 10 drops in a spoonful of sweetened water after the meals). This medication is to be kept up for several weeks.

"Electricity gives undoubtedly good results in this affection. Few therapeutists, however, (like Duchenne), advocate faradization; the majority (among these Eulenburg, Huchard, Meyer, Remak, and Ziemssen) prefer galvanization. Ziemssen has directly proven, by experiments, that the constant current influences the heart like the striped muscles, and that the effects of the current, if applied to the thorax walls, are just the same as if applied to the heart directly. Ziemssen obtained wholly negative results from faradization. The application of the current can be effected in various manners. Ziemssen recommends to place one electrode to the sternum and the other to the back, at a height corresponding to the position of the heart. Eulenburg places the anode over the heart, and the kathode like Ziemssen, or the anode to the sternum and the kathode to the neck. Erb advocates the same method.

"In the patients treated by myself I have invariably placed the anode on the neck, in the region of the nerve-trunks, and the kathode on the apex. After a couple of minutes I could always notice a considerable decrease in the frequency of the apex-beat (10 to 30).

"After the application the patients usually pronounced their condition improved. Eminently successful results could be obtained in the paroxysms returning several times daily, a few faradic applications usually sufficing to either remove the paroxysms wholly or to lessen their severity. Satisfactory results were likewise obtained in intercostal neuralgia and cardialgia.

"It is possible that the great success of galvanization obtained in my patients is partly to be attributed to the simultaneous use of medicinal waters (chiefly iron), which I invariably prescribe in this affection. It is an indisputable fact that iron exercises a tranquillizing influence upon the action of the heart, not only in nervous palpitations, but also in developed cardiac defects, and even in incomplete compensation.—*Therapeutic Gazette.*

